# Personal

**Published:** 1903-05

**Authors:** 


### PERSONAL.

Dr. D. W. Harrington, of Buffalo, who has been in the South for some weeks on account of impaired health, has returned and is reported as much improved.

Hon. James O. Putnam, one of the most distinguished citizens of Buffalo, who suffered from a paralytic seizure some weeks ago, is improved, having recovered consciousness, speech and the complete use of his faculties. He is reported as progressing favorably toward recovery.

Dr. Augustus Pohlman, an instructor of Cornell University, son of Dr. Julius Pohlman of Buffalo, has returned from Germany where, during the past two years, he has been pursuing anatomical and other studies.

Dr. E. E. Blaauw, of Buffalo, recently entertained guests at dinner at the Iroquois in honor of Professor Carl G. Eigenmann, of the University of Indiana.

Dr. A. S. Freeman, of Buffalo, the veteran dentist, was tendered a complimentary dinner at The Iroquois, March 31, 1903, to commemorate the 40th anniversary of his dental practice in this city. It was attended by about 100 dentists and was presided over by Dr. C. W. Stanton, who acted as toastmaster. Dr. Freeman's speech was filled with reminiscences of the earlier years of his practice. Among the out-of-town guests present were: Drs. Frank French, R. H. Hofheinz, W. W. Smith, J. E. Line, Rochester; W. A. White, Phelps; G. W. Hoystradt, Ithaca; J. Granger, Mayville; A. M. Wright, Troy; and C. H. Sharp, Lockport.

Dr. and Mrs. Charles Cary, of Buffalo, sail for Europe early in May, to spend a few weeks in Continental travel.

Dr. Henry L. Elsner, of Syracuse, delivered an address at Elmira N. Y., on "Medicine of fifty years ago and of today," on the occasion of the celebration of the 50th anniversary of the Elmira Academy of Medicine, April 1, 1903.

Dr. Benjamin Franklin Rogers, of Buffalo, has removed his office from No. 221 Franklin Street to 222 Franklin Street, just opposite, where he will continue the practice of ophthalmology. Hours: 9 to 1 and 3 to 4.

---

Dr. W. G. Macdonald, of Albany, read a paper before the section on surgery of the Buffalo Academy of Medicine, Tuesday evening, April 7, 1903, the subject of which was "The rôle of cholecystitis in acute inflammations of the peritoneum, with report of cases." It was an admirable and scholarly presentation of a topic of most absorbing interest. The diction and rhetoric of the paper should serve as a model to stimulate others to careful preparation, when presenting themes before medical societies.

---

Dr. Edgar A. Forsyth, of Buffalo, has removed from 326 Franklin Street to 322 Franklin Street, where he will continue the practice of laryngology. Hours: 9 to 1 and 7 to 8. Both telephones.

---

Dr. Nelson W. Wilson, of Buffalo, sanitary officer of the Pan-American Exposition, was recently invited to St. Louis, to give the benefit of his experience in the sanitary management of the Buffalo exposition to the management of the Louisiana Purchase Exposition. We expect to print something from Dr. Wilson's facile pen, touching his observations of the St. Louis Fair.

---

Dr. William G. Taylor, of Buffalo, has received an appointment to the medical staff in the children's department of St. Mary's Infant Asylum in this city.

---

Dr. William Meisburger, of Buffalo, has removed from No. 355 Genesee Street to No. 891 Michigan Street.

---

Dr. Montgomery A. Crockett, of Buffalo, who has recently spent some time in the South during convalescence from a severe illness, has returned fully recovered and resumed his professional practice and teaching. His office and residence are at 452 Franklin Street.

---

Dr. William C. Krauss has been reappointed by Governor Odell a member of the Board of Visitation for the Buffalo State Hospital.

---

Dr. Herman E. Hayd, of Buffalo, has been elected surgeon to the Buffalo German Hospital, vice Herman Mynter, deceased.

Dr. Thomas B. Carpenter, of Buffalo, has removed from No. 142 North Pearl Street to No. 533 Franklin Street. Hours: 3 to 4 and 7 to 8. Telephone, Tupper 304.

Professor von Mikulicz, of Breslau, the renowned German surgeon, visited Buffalo, April 25, 1903, and was the guest of honor at a complimentary dinner tendered him by representative members of the medical profession of this city. The dinner was served at the Iroquois, about thirty covers being laid. The occasion was full of pleasantry and delightful incidents and will be long remembered by the participants. The special object of Professor von Mikulicz's visit to this country is to take part in the proceedings of the congress of American Physicians and Surgeons, at Washington, May 12-14, 1903, where is scheduled to discuss the surgery of the pancreas.

Dr. Charles O.Chester, of Buffalo, has removed from No. 217 to No. 326 Franklin Street. He has purchased the last-named property and will continue the practice of laryngology permanently at that number. Hours, 9-1, and 7 p.m.. Both telephones.

Sir James Grant, of Ottawa, Can., has recently celebrated his jubilee as a member of the medical profession by a dinner and a smoking concert, with which he entertained his friends in St. Andrew's Hall, February 4, 1903. Sir James, who is one of the most prominent members of the Canadian profession, is the father of Dr. Henry Y. Grant, of Buffalo.

Hon. Charles S. Francis, of Troy, has been elected by the legislature a regent of the University of the State of New York, vice Hon. Martin I. Townsend, deceased.

